# Supplementary material for: A Systematic Review of High-Dose Methotrexate for Adults with Primary Central Nervous System Lymphoma
Source: Cancers (Basel). 2023 Feb 25;15(5):1459. doi: 10.3390/cancers15051459 (PMC10000886; doi:10.3390/cancers15051459)
Supplement: Supplementary file 1 [file cancers-15-01459-s001.zip › cancers-2211501-supplementary.pdf]

Supplementary Materials

Table S1. Mean or median age of patients included in the clinical trials

Author (year)	Cohort number	Mean age (SD), years	Median age, years	Age range, years	N
Batchelor et al. (2003)	125	59.8 (12.5)		47 – 72	25
Bromberg et al. (2019)	256		61	55 – 67	199
Columbat et al. (2006)	253		52	21 – 60	25
DeAngelis et al. (1992)	126		58	29 – 79	31
DeAngelis et al. (2002)	60		56.5		98
Ferrari et al. (2016)	257		57.5	50 – 64	219
Ferrari et al. (2006)	128		44	18 – 70	41
Ferrari et al. (2009)	261		59	25 – 74	79
Fritsch et al. (2017)	67		74.5	66 – 85	107
Fu et al. (2021)	259	58.3 (10.1)		47 – 69	108
Ghesquières et al. (2010)	260		63	20 – 82	99
Glass et al. (2016)	130		57	24 – 73	53
Hoang-Xuan et al. (2003)	77		72	60 – 81	50
Illerhaus et al. (2009)	82		70	57 – 79	30
Illerhaus et al. (2016)	81		41.5	18 – 65	79
Korfel et al. (2015)	262	61.5 (11.1)		50 – 72	410
Laack et al. (2011)	223		51.5	34 – 69	36
Morris et al. (2013)	135		60	30 – 79	52
O'Brien et al. (2000)	136		58	25 – 76	46
Olivier et al. (2014)	238		65	60 – 71	35
Omuro et al. (2015)	105		45	23 – 67	32
Omuro et al. (2015)	264		72	60 – 85	95
Pels et al. (2003)	108		51	27 – 75	65
Salamoon et al. (2013)	249		52	20 – 65	40
Shah et al. (2007)	115		53	30 – 76	30
Wieduwilt et al. (2012)	165		62.5	40 – 84	31

Table S2. Leucovorin rescue doses for study cohorts

Author (year)	Cohort number	HDMTX dose (g/m^2)	Leucovorin (LV) rescue, Dose and frequency	LV rescue - Time of initiation (hours after HDMTX infusion)
Batchelor et al. (2003)	125	8	Supportive care with LV, NOS	24
Bromberg et al. (2019)	256	3	Supportive care with LV, NOS	No data
Columbat et al. (2006)	253	3	Supportive care with LV, NOS	No data
DeAngelis et al. (1992)	126	1	10 mg Q6 hours	No data
DeAngelis et al. (2002)	60	2.5	20 mg Q6 hours	24
Ferrari et al. (2016)	257 A,B,C	3.5	15 mg/m^2 Q6 hours	24
Ferrari et al. (2006)	128	3.5	No data	No data
Ferrari et al. (2009)	261 A,B	3.5	Supportive care with LV, NOS	No data
Fritsch et al. (2017)	67	3	No data	No data
Fu et al. (2021)	259 A,B	3	Supportive care with LV, NOS	12
Ghesquières et al. (2010)	260 A,B	1.5	No data	No data
Ghesquières et al. (2010)	260 C	3	No data	No data
Glass et al. (2016)	130	3.5	25 mg Q6 hours	24
Hoang-Xuan et al. (2003)	77	1	25 mg Q6 hours	24
Illerhaus et al. (2009)	82	3	15 mg/m^2 Q6 hours	24
Illerhaus et al. (2016)	81	8	No data	No data
Korfel et al. (2015)	262 A,B	4	No data	No data
Laack et al. (2011)	223	1.5	No data	No data
Morris et al. (2013)	135	3.5	100 mg/m^2 Q6 hours	No data
O'Brien et al. (2000)	136	1	15 gm Q6 hours	24
Olivier et al. (2014)	238	3	50 mg Q6 hours	24
Omuro et al. (2015)	105	3.5	Supportive care with LV, NOS	No data
Omuro et al. (2015)	264 A,B	3.5	Supportive care with LV, NOS	No data
Pels et al. (2003)	108	5	30 mg/m^2	10
Salamoon et al. (2013)	249	3	No data	No data
Shah et al. (2007)	115	3.5	20-25 mg Q6 hours	24
Wieduwilt et al. (2012)	165	8	Supportive care with LV, NOS	No data

NOS, not otherwise specified
